# Repair of thumb defect by using the toenail flap: biomechanical analysis of donor foot—a retrospective cohort study

**DOI:** 10.1186/s13018-019-1330-7

**Published:** 2019-09-02

**Authors:** Chunjie Liu, Lei Liu, Guoli Liu, Siyu Tian, Jiangbo Bai, Kunlun Yu, Dehu Tian

**Affiliations:** 1 Department of Orthopedics, Tangshan Workers Hospital, Tangshan, 063000 Hebei People’s Republic of China; 2 Department of Orthopedics, Changping District Hospital, Beijing, 102200 People’s Republic of China; 3 Department of Orthopedics, The Second Hospital Of Tangshan, Tangshan, 063000 Hebei People’s Republic of China; 4 Department of Hand Surgery, The Third Affiliated Hospital Of Hebei Medical University, Shijiazhuang, 050017 Hebei People’s Republic of China

**Keywords:** Amputation, Thumb reconstruction, Toe transfer, Foot morbidity, Biomechanics

## Abstract

**Background:**

The thumb accounts for 50% of the total hand function. This study reports the functional outcomes and complications of people with traumatic thumb amputations who underwent toe-to-thumb reconstruction.

**Methods:**

From January 2013 to January 2018, 29 patients with second-degree thumb defect underwent thumb reconstruction with distal phalangeal braided toenail flap. The footscan foot pressure gait analysis system was used to measure the index changes of the same foot before and after 1, 3 and 6 months. The contact area, peak pressure, impulse value, contact time of each gait phase, centre of gravity coordinate and foot balance were analysed statistically.

**Results:**

Twenty-nine cases of thumb reconstruction recovered well. After following up for 6–15 months, the appearance of the reconstructed thumb was close to normal, and the sensation was restored to S3^+^. The two-point discrimination was 6–8 mm, and the function of the thumb was good. The function of the donor foot was well restored, and no skin ulceration, pain and claudication were noted during walking. Compared with that before the operation, the biomechanical indices of the donor foot were basically restored to normal 6 months after the operation. Only the stress and impulse values of the third metatarsal head were significantly increased, forming a stress concentration area centred on the third metatarsal head.

**Conclusions:**

This study confirmed that the toenail flap with distal phalangeal bone restored the second-degree thumb defect without destroying the main functional structure of the sole. The biomechanical indices of the donor foot were basically restored to normal 6 months after the operation. Only the stress concentration area centred on the third metatarsal head, and the pain on the forefoot was induced after the operation. Discomfort, callus formation, metatarsal fasciitis, etc., can lead to fatigue fracture of the third metatarsal bone in severe cases, which requires further follow-up and observation.

**Trial registration:**

Clinicaltrials.gov, NCT03879941; registered on 10 March 2019, retrospectively.

## Background

Traumatic thumb defect is a common injury in hand surgery. The thumb function accounts for 50% of the total hand function [1]. The pinching, grasping and gripping functions of the hand are mostly lost, which seriously affects the function of the hand. Hence, its reconstruction after a defect is very necessary. An ideal reconstituted thumb should have moderate length, stable stent, strong activity, normal feeling and well-proportioned shape. With the development and improvement of microsurgical technology, restoration has developed from simple extensive reconstruction to refined modified reconstruction to obtain the ideal function and shape of the reconstructed thumb.

Many traditional repair methods for the treatment of second-degree thumb defects are available, such as lengthening the stump, repairing the skin tube after iliac bone grafting and reconstructing the thumb with free second toe transplantation [2–4]. However, the appearance of the thumb is different from that of the normal thumb, which does not satisfy the patients. Compared with the common toe transplantation for thumb reconstruction, the toenail flap with the distal phalangeal bone does not sacrifice the toe, has less influence on the function of the foot and is close to the normal appearance of the thumb. Since Morrison et al. [5] first used the free toenail flap to wrap the tibial block for thumb reconstruction in 1980, the technique has been widely used clinically. After transplantation, the skin in the load-bearing area is not wear-resistant, lacks soft tissue protection and is close to the toe bone; thus, the donor toe must not bear weight. Repeated friction causes erosion of the skin grafting area and leads to the long-term formation of a thick callus, affecting the weight of the donor foot and normal walking, and plantar stress distribution changes [6]. Therefore, considerable attention has been paid to the effect of donor foot function. Many scholars believe that the success criterion of toenail flap transplantation should not only be the survival of the flap itself but also the effect on the feeding function.

At present, literatures about the biomechanical analysis of the donor foot after thumb reconstruction with distal phalangeal flap are few. The plantar dynamic biomechanical analysis can reflect the dynamic changes of compressive stress, impulse and centre of gravity in the area of plantar contact in a comprehensive and accurate way, which is significant for understanding the biomechanical changes of the donor foot [7]. From January 2013 to January 2018, 29 patients with second-degree thumb defect underwent thumb reconstruction by free transplantation of toenail flap with distal phalangeal bone. The footscan® foot pressure gait analysis system was used to analyse the dynamic biomechanics of the foot before and after the operation.

## Methods

### Study cases

**Fig. 1:**
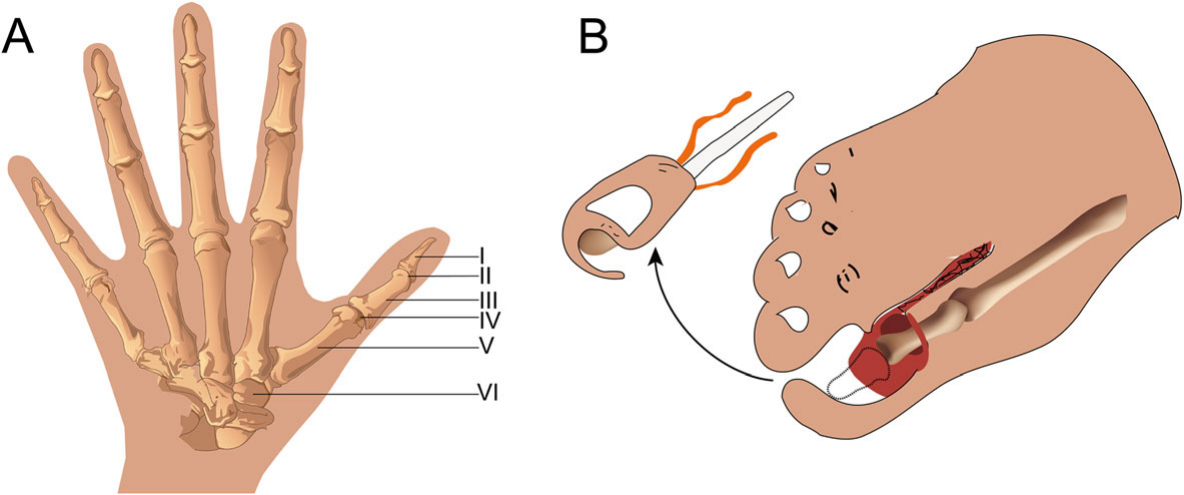
**a** The range of thumb defect. **b** A schematic diagram of the toenail flap with distal phalanx

This work has been reported in line with the STROCSS criteria [8]. From January 2013 to January 2018, a total of 29 patients were recruited according to the criteria described in Table 1 (Fig. 1a). The causes of injury were as follows: 2 cases of amputation and loss, 21 cases of machine rolling damage, 5 cases of rotational tear and replantation failure and 1 case of childhood traumatic defect. The patients included 19 males and 10 females with age of 17–46 years (mean 29.1 years), height of 156–179 cm (average 170.4 cm), weight of 50–80 kg (average 62.3 kg) and foot length of 23–26 cm (average 24.5 cm). The defect cases included 12 on the left side of the toe and 17 on the right side. The follow-up time was 6–15 months. After the operation, the foot incision healed by first intention. Before treatment, informed consent was obtained from the patients and their families.

**Table 1:** List of inclusion and exclusion criteria of the study

Inclusion criteria	Exclusion criteria
·Patients with second-degree thumb defect [9]	·Patients with vascular injuries requiring revascularization
·Only one injured hand	·Concomitant phalanx fractures or other injuries needing immobilization
·Without any foot disease or deformity before injury	·Smokers
·Written informed consent to undergo the surgical procedure	·Uncompensated diabetes, neoplasia, haemocoagulative alterations, psychic disorders

### Surgical method

According to the wound area, the range of the toenail flap was designed, and the peroneal blood vessels of the toenail were reserved. If necessary, the dorsal flap of the foot was taken. According to the flap design, an ‘S’-shaped incision was made on the lateral side of the foot and the proximal side of the flap. The skin was cut, and subcutaneous separation was performed. The dorsal vein and the armour flap usually have 2–3 branches. The skin on the back of the toe and flap was cut along the temporal side of the flap, and the veins from the dorsal foot veins to the tibial side of the second toe were dissected. The fibular side of the toe and the second tibial proper artery, nerve and bottom artery were separated between the toes. The skin around the armour flap was cut, freeing the flap; then, the skin around the second toe flap was cut, freeing the second toe flap. A combined flap pedicled with the dorsal plantar vein and the first dorsal metatarsal artery (or the digital artery) and the tibial flap of the second toe were formed by cutting off the interphalangeal joint. The donor area was directly sutured or taken with a full-thickness skin graft. After the recipient area was ready, the pedicle was broken according to the length of the vascular pedicle needed by the recipient area. The toenail flap covered the dorsal side of the thumb; then, interphalangeal fusion and Kirschner wire fixation were performed. The second toe flap covered the palm of the thumb, and the skin between the two flaps was loosely sutured. The toe nerves on both sides of the flap were sutured with the finger nerves on both sides of the thumb. The dorsal cutaneous nerve of the foot was sutured with the superficial branch of the radial nerve. The first dorsal artery (or toe bottom artery) was anastomosed to the radial branch of the radial artery, and the dorsal vein of the foot was anastomosed to the cephalic vein. On the radial side of the thumb, two skin defects were found in the trigonometric region between the skin flaps. Skin graft with full-thickness was recommended (Fig. 1b).

**Fig. 2:**
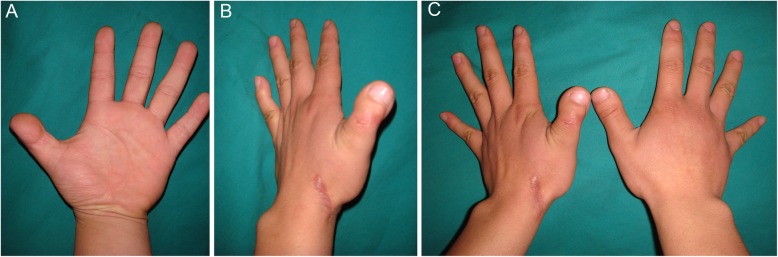
A 25-year-old male patient. The left thumb was amputated at the interphalangeal joint level. A toenail flap with distal phalanx was transferred to reconstruct the thumb. **a**, **b** Follow-up photograph 1 year postoperatively shows a good replica of the intact thumb. **c** Comparison of appearance with healthy thumb

**Fig. 3:**
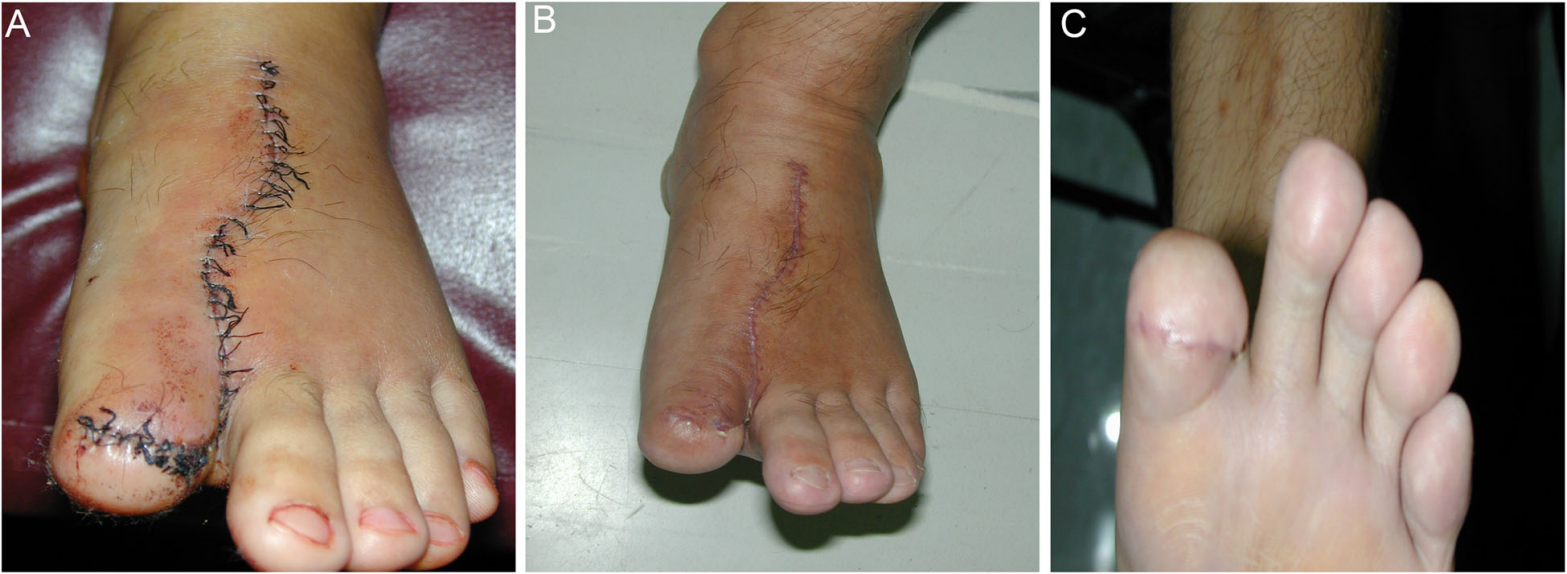
The same patient as in Fig. 2. **a** Foot incision. **b** Dorsal appearance of donor foot 1 year postoperatively. **c** Plantar appearance of the donor foot

Twenty-nine cases of thumb reconstruction recovered well. After follow-up for 6–15 months, the appearance of the reconstructed thumb was close to normal, and the sensation was restored to S3^+^. The two-point discrimination was 6–8 mm, and the function of the thumb was good. According to the hand function evaluation criteria of the Hand Surgery Society of the Chinese Medical Association [10], 21 cases were found to be excellent, and 8 cases were rated as good (Fig. 2). The function of the reconstructed thumb was well restored, and no skin ulceration, pain and claudication occurred during walking (Fig. 3).

### Test system

Plantar pressure gait analysis system produced by footscan Co., Belgium, was used. The test plate is 10 m long and 40 cm wide, and the measuring area is 2 m in the centre of the plate.

### Test method

**Fig. 4:**
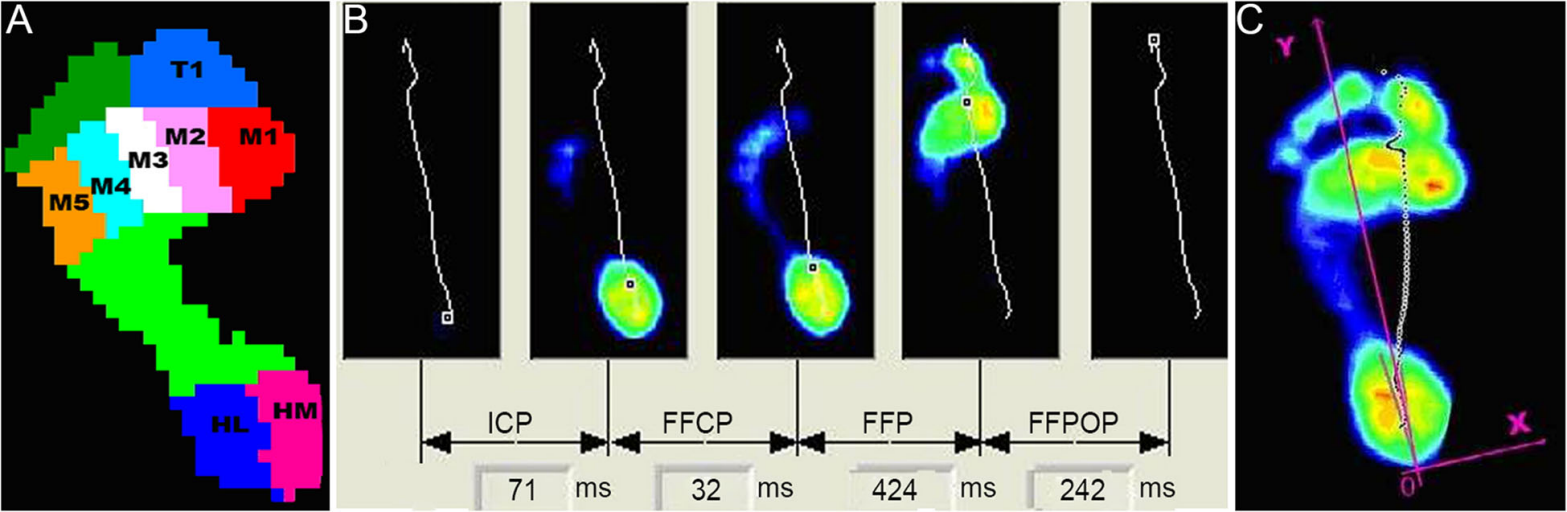
Gait analysis was performed using F-Scan system (Footscan software 7.0 Gait, RsScan International). **a** The location of eight anatomic important areas of the peak pressure footprint. **b** Four phases of the total foot contact. **c** The *x*-component and *y*-component of the centre of the pressure. The *x*-component is positive when positioned medially to the heel-M2/3 axis and negative when it is positioned. A normal case (before the operation)

According to the anatomical region, the plantar was divided into eight stress regions: medial heel (HM), lateral heel (HL), first metatarsal head (M1), second metatarsal head (M2), third metatarsal head (M3), fourth metatarsal head (M4), fifth metatarsal head (M5) and first toe area (T1) (Fig. 4a). For a specified lower limb, the activity of a gait cycle can be divided into two phases: supporting and swing phases. The supporting phase is divided into initial contact phase (ICP), forefoot contact phase (FFCP), foot flat phase (FFP), forefoot push-off phase (FFPOP) and other action phases (Fig. 4b). The ICP starts from the beginning of the foot to the first humeral head touching the ground. The FFCP starts from the first humeral head to the entire humeral head touching the ground. The FFP starts from the entire metatarsal bone touching the ground to the heel starting to leave the ground. The FFPOP is the moment when the heel and foot leave the ground. The centre of the heel posterior margin is the origin of plantar 0, and the line between the origin and the second and third phalanges is the *y*-axis. The axis perpendicular to the origin is the *x*-axis. The arrow of the *x*-axis pointing to the inside is positive. The distribution of centre of plantar stress can be measured quantitatively by this coordinate (*x*, *y*) (Fig. 4c). Foot balance refers to the difference of compressive stress between the inside and the outside of the foot at a certain time. Its formula is (FHM + FM1 + FM2) − (FHL + FM4 + FM5).

The subjects walked on the test plate naked (walking speed 95–117 steps/min, step 60–80 cm). The subjects felt no pain or discomfort during the whole test. The distribution of plantar compressive stress was obtained when walking flat. Generally, the system moves back and forth 3–4 times and automatically takes the average value. The data are collected into the computer image analysis system, which then performs measurement and analysis of the shape and time. Given the great difference of the stress distribution of the sole between normal people, especially in the walking state, the stress distribution of the sole was affected by wearing shoes, walking posture, foot shape and walking speed. We measured the changes of the index of the same foot, including the various parts of the sole by self-control, before and after 1, 3 and 6 months. The contact area, peak pressure, impulse value, contact time of each gait phase, centre of gravity coordinate and foot balance were analysed statistically.

### Statistical analysis

All measurement data were analysed using SPSS 22.0. The data were processed by statistical software. The analysis of variance of repeated measurements was used before and after the operation. The LSD-t method was used in the comparison between groups. *P* < 0.05 was considered to be statistically significant.

## Results

**Fig. 5:**
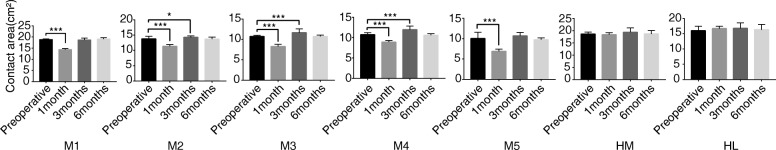
Compared with that before the operation, the contact area of the donor foot in M1–M5 and M2–M4 areas decreased significantly 1 and 3 months after the operation, respectively. No significant change was observed 6 months after the operation. *P < 0.05, ***P < 0.001; *N* = 29. *P* < 0.05 was considered significant

Compared with that before the operation, the contact area of the donor foot in M1–M5 and M2–M4 areas decreased significantly 1 and 3 months after the operation, respectively, and the difference was statistically significant (*P* < 0.05). No significant change was observed 6 months after the operation (Fig. 5).

**Fig. 6:**
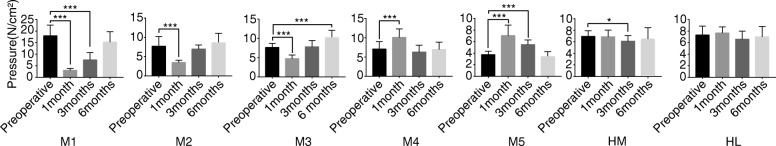
Compared with that before the operation, the maximum pressure of M1–M3 decreased and that of M4 and M5 increased 1 month after the operation. Three months after the operation, the pressure of M1 decreased, and that of M5 increased. Six months after the operation, the pressure of M3 increased. The difference was statistically significant. *P < 0.05, ***P < 0.001; *N* = 29. *P* < 0.05 was considered significant

Compared with that before the operation, the maximum pressure of M1–M3 decreased and that of M4 and M5 increased 1 month after the operation. Three months after the operation, the pressure of M1 decreased, and that of M5 increased. Six months after the operation, the pressure of M3 increased. The difference was statistically significant (*P* < 0.05) (Fig. 6).

**Fig. 7:**
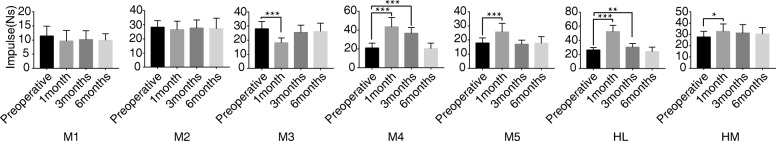
One month after the operation, the impulse value of M3 decreased and those of M4, M5 and HL increased. However, 3 months after the operation, the impulse values of M4 and HL increased. The difference was statistically significant (*P* < 0.05). No significant change was observed 6 months after the surgery. *P < 0.05, **P < 0.01, ***P < 0.001; *N* = 29. *P* < 0.05 was considered significant

One month after the operation, the impulse value of M3 decreased and those of M4, M5 and HL increased. However, 3 months after the operation, the impulse values of M4 and HL increased. The difference was statistically significant (*P* < 0.05). No significant change was observed 6 months after the surgery (Fig. 7).

**Fig. 8:**
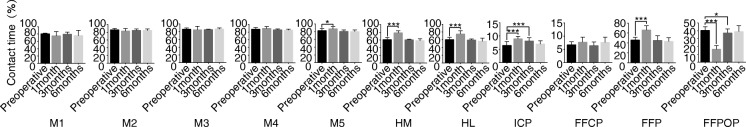
One month after the operation, the contact time of FFPOP decreased, whereas those of ICP, FFP, M5, HM and HL increased. After 3 months, the contact time of ICP increased, whereas that of FFPOP decreased. The difference was statistically significant. *P < 0.05, ***P < 0.001; *N* = 29. *P* < 0.05 was considered significant

One month after the operation, the contact time of FFPOP decreased, whereas those of ICP, FFP, M5, HM and HL increased. After 3 months, the contact time of ICP increased, whereas that of FFPOP decreased. The difference was statistically significant (*P* < 0.05) (Fig. 8).

**Fig. 9:**
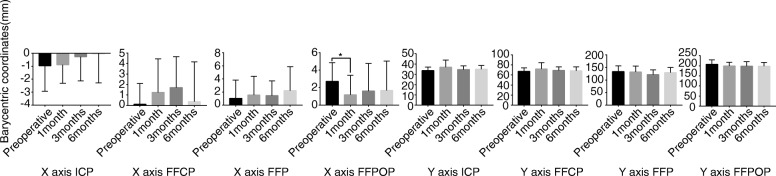
A statistically significant difference was observed in the *x*-coordinate value of FFPOP 1 month after the operation. *P < 0.05, *N* = 29. *P* < 0.05 was considered significant

A statistically significant difference was observed in the *x*-coordinate value of FFPOP 1 month after the operation (*P* < 0.05) (Fig. 9).

**Fig. 10:**
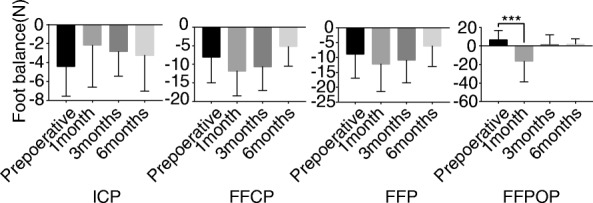
The lateral pressure of the foot in the FFP and FFPOP periods increased 1 month after the operation, and the difference was statistically significant (*P* < 0.05). No significant change was observed in other comparisons. ***P < 0.001, *N* = 29. *P* < 0.05 was considered significant

The lateral pressure of the foot in the FFP and FFPOP periods increased 1 month after the operation, and the difference was statistically significant (*P* < 0.05). No significant change was observed in other comparisons (Fig. 10).

## Discussion

The thumb accounts for 50% of the total hand function; hence, any thumb defect should be reconstructed. There are many traditional repair methods for second-degree thumb defects; however, the appearance of the reconstructed thumb is different from that of the normal thumb, causing dissatisfaction amongst patients. The toenail flap with the distal phalangeal bone does not sacrifice the toe, has less influence on the function of the foot and is close to the normal appearance of the thumb [11]. At present, literatures about the biomechanical analysis of the donor foot after thumb reconstruction with distal phalangeal flap are few.

When the forefoot is loaded, the load-bearing pressure passes through the metatarsal bones of the transverse arch of the foot to the central metatarsal bones, the distance between the metatarsal bones increases and the transverse arch of the foot sinks. The kinetic energy of the impact force is transformed into the elastic deformation potential energy of the transverse arch through the deformation of the transverse arch of the foot [12, 13]. The transverse arch of the foot is a roof-like upward and forward arc load-bearing structure. The medial load-bearing focus is the first metatarsal bone, and the lateral load-bearing focus is the fifth metatarsal bone. The first metatarsal bone bears more weight of up to 50% when the heel is slightly off the ground. A majority (82%) of normal individuals have a static peak foot pressure at the heel, followed by the metatarsal region, which lessens the stress on the toes. Most of the dynamic peak foot pressure is located in the first and second metatarsal bones. That is, the maximum pressure of the foot moves forward whilst walking. The pressure on the area of the forefoot metatarsal bones is the largest, and the first toe becomes the main force position. In addition, compared with standing time, the pressure changes in different parts of the foot during walking are also different [14, 15].

The footscan foot pressure gait analysis system has been widely used in clinical disease research. It can measure the stress distribution of the foot during travelling and has good reliability and repeatability. Horizontal gait is the most basic and important function of the foot. The foot function changes are caused by many pathological changes. Static plantar stress testing may not show abnormalities, but dynamic stress testing can. The stress abnormalities in a certain area are large and small, or the stress intervals are shortened or prolonged [16]. Amongst them, the dynamic peak pressure refers to the peak stress when the sole touches the ground. Its increase indicates that the pressure in the unit area increases when the sole touches the ground. Impulse value refers to the product of touchdown time and pressure in each stress area of the foot in each cycle. The increase of impulse is more suggestive of fatigue damage and subsequent foot deformities, such as valgus, than the increase of peak stress [17]. Therefore, this clinical experiment evaluates the regularity of the stress distribution of the plantar with time during a walking cycle from a dynamic perspective.

Normally, M1 has the largest weight-bearing ratio amongst the five metatarsal heads, whether in static loading or walking. When the heel is just off the ground, the forefoot is loaded, and the first metatarsal bone bears approximately 50% of the total metatarsal pressure. When the forefoot rolls forward perpendicular to the transverse arch during the weight-bearing walk, the load begins at the two metatarsal bones and gradually shifts to the central metatarsal bones. The plantar stress reaches a dynamic balance. When the foot is fully raised, the load ratio of M1 is 32% [18]. After the operation, because the first toe became shorter and the force arm was too short, the gravity could not be transmitted to the ground along M1 and the first toe during the propulsive period; hence, the stress value under M1 was significantly reduced (significantly different from the normal group). The triangular load-bearing structures of the first toe and the first and second metatarsal bones were changed, and the load was shifted outward, resulting in the compensatory increase of stress under the fourth and fifth metatarsal bones. At the same time, given the pain and scar of the first toe wound within 1 month after the operation, the body prevented forefoot landing, reduced the M1–M5 contact area and shortened the FFP and FFPOP phases of forefoot landing, thereby causing the prolongation of hind foot landing time. The ICP period, which reflects the landing time of the hindfoot, and the contact time of HM and HL were prolonged.

Three months after the operation, the contact area of M2–M4 increased, the pressure of M1 decreased, the pressure of M5 increased, the values of M4 and HL increased, the contact time of ICP increased and the contact time of FFPOP decreased. Three months after the operation, the compressive stress of the first toe disappeared (part of the first toe had been transplanted), the stress value of the first metatarsal bone decreased significantly and the stress value of the fifth metatarsal bone increased compensatively. This is because normal gait movement requires the first metatarsophalangeal joint to flex from 65 to 75° and the toe to be fixed on the ground to carry the gravity transmitted through the first metatarsal bone. When the final heel is fully elevated, a triangular weight-bearing structure consisting mainly of the first toe and the first and second metatarsal bones is formed. The lateral metatarsal bones and toes only play an auxiliary role because the first toe conducting the gravity of the first metatarsal is partially removed; hence, the gravity of the first metatarsal can no longer be fully borne by the first metatarsal. The body protects the first metatarsal from compressive stress, compensates the fifth metatarsal for compressive stress and increases the lateral compressive stress and impulse of the foot by reducing the medial contact time of the forefoot. The impulse of M4 and HL increased, the contact time of ICP increased and the contact time of FFPOP decreased; thus, the first metatarsal area after surgery does not bear too much load.

Six months after the operation, the results showed that all the indices were basically restored to normal; only the stress and impulse values of M3 were significantly increased. Especially during the advancing period of gait, the gravity mainly travelled along M3 and the third toe to the ground, forming a stress concentration area centred on M3, and the pain on the forefoot was induced after the operation. Discomfort, callus formation, metatarsal fasciitis, etc., can lead to fatigue fracture of the third metatarsal bone in severe cases [19], which requires further follow-up and observation.

We acknowledge limitations of this retrospective study. First, the number of patients is small. In our selected cases, the cause of second-degree thumb defect included almost all common hand injuries, which were widely representative and were absolute indications for thumb reconstruction by using the toenail flap with distal phalanx. An analysis of the parameters related to donor foot after thumb reconstruction with free second toe transplantation was reported [20]. However, to our knowledge, there is no published study that specifically reported the biomechanical analysis of the donor foot after partial toe transplantation. Second, shorter follow-up period may overestimate the expected effect. Our research has confirmed that the biomechanical indexes of the donor foot have basically returned to normal after 6 months. Long-term observation remains to be further studied.

## Conclusions

This study confirmed that the toenail flap with distal phalangeal bone restored the second-degree thumb defect without destroying the main functional structure of the sole. The biomechanical indices of the donor foot were basically restored to normal 6 months after the operation, and the donor foot was uncomfortable. It is worthy of promotion and application in clinics.

## Data Availability

The datasets used and/or analysed during the current study are available from the corresponding author on reasonable request.

## Abbreviations

FFCP: Forefoot contact phase
FFP: Foot flat phase
FFPOP: Forefoot push-off phase
HL: Lateral heel
HM: Medial heel
ICP: Initial contact phase
M1: First metatarsal head
M2: Second metatarsal head
M3: Third metatarsal head
M4: Fourth metatarsal head
M5: Fifth metatarsal head
T1: First toe area
